# Transvaginal Ultrasound in Fertile Patients with Suspected Appendicitis: An Experience Report of Current Practice

**DOI:** 10.1155/2012/481797

**Published:** 2012-03-15

**Authors:** Malek Tabbara, Nikolaos Evangelopoulos, Luigi Raio, Vanessa Banz, Heinz Zimmermann, Corinne Kim-Fuchs, Aristomenis K. Exadaktylos

**Affiliations:** ^1^Department of Emergency Medicine, Inselspital, 3010 Bern, Switzerland; ^2^Department of Gynaecology and Obstetrics, Inselspital, 3010 Bern, Switzerland; ^3^Department of Visceral Surgery and Medicine, Inselspital, 3010 Bern, Switzerland; ^4^Division of Anaesthesia, Intensive Care and Emergency Medicine, Department of Emergency Medicine Inselspital, 3008 Bern, Switzerland

## Abstract

*Background*. Transvaginal ultrasound (TVU) in female patients with acute right lower quadrant (RLQ) abdominal pain is time and infrastructure intensive and not always available. This study aims to evaluate the role of TVU in these patients. *Methods*. Retrospective analysis identified 224 female patients with RLQ pain and TVU. *Results*. TVU revealed an underlying pathology in 34 (15%) patients, necessitating a diagnostic laparoscopy in 12 patients. Six patients (2%) had a true gynaecological emergency. The remaining 23 patients did not require surgery. The other 190 patients with RLQ pain had a bland TVU; 127 (67%) were discharged, while 63 patients (33%) received a diagnostic laparoscopy. *Conclusion*. The incidence of true gynaecological emergencies requiring urgent surgical intervention is very low in our patient cohort. TVU is a helpful tool if performed by a physician who is well trained in TVU.

## 1. Introduction

Evaluating acute right lower quadrant (RLQ) abdominal pain in nonpregnant female patients (NPF) remains a clinical challenge despite advances in—and more liberal use of—imaging techniques. Workup of this patient group is more difficult than with male patients, due to the local anatomical differences with many gynaecological pathologies potentially mimicking more common diseases such as acute appendicitis.

Although this symptom is very common in emergency departments (EDs), there is no clear literature providing a thorough and systematic approach that should be followed by ED physicians when facing RLQ abdominal pain in NPF. While diagnostic tools such as abdominal computed tomography and/or magnetic resonance imaging can improve diagnostic accuracy [1, 2], the problem of unnecessary exposure to radiation and/or the lack of 24-hour availability remains a real issue. Moreover, these high-resolution studies can remain inconclusive in the absence of radiology staff to interpret them. The role of transvaginal ultrasound (TVU) remains inadequately documented in the literature. A relatively recent study by Tayal et al. showed that TVU performed by ED physicians could increase the diagnosis of gynaecological problems and ultimately change diagnostic decisions by physicians—in NPF with undifferentiated RLQ pain [3]. Nevertheless, TVU remains an invasive examination and requires trained physicians. A study by Bennett and Richards showed that the majority of women considered that TVU is an uncomfortable procedure [4]. 

Furthermore, as most of the urgent TVUs in the European setting are performed outside the ED, this service is potentially time consuming and may not always be available. The absence of consensus among ED physicians on how to evaluate NPF presenting with RLQ abdominal pain may lead to a delay in diagnosis and unnecessary use of resources and diagnostic tools. This may result in unnecessary costs and an invasive and uncomfortable examination for the patients. The aim of this study is to review our ED experience in approaching NPF with RLQ abdominal pain and to describe the role of TVU in supporting decisions made by ED physicians.

## 2. Methods

We identified 224 female patients admitted to our university Hospital ED between 2000 and 2009 with acute right lower quadrant abdominal pain in which appendicitis was a very likely differential diagnosis, and who underwent a TVU. The electronic medical records of these patients were reviewed using “Qualicare” (Qualidoc AG, Trimbach, Switzerland, http://www.qualidoc.ch/), and the following data were collected: general patient demographics, prior history of sexually transmitted disease, abdominal or gynaecological surgery, previous pregnancies, physical exam, TVU findings, final diagnosis, need for surgery, and intraoperative findings. We excluded patients who (1) were younger than sixteen years of age, (2) were currently pregnant, (3) were postmenopausal “nonfertile” patients (4) had undergone an abdominal or gynaecological procedure within the preceding two weeks, (5) had a history of right oophorectomy/salpingectomy, (6) had a history of appendectomy, and (7) were haemodynamically unstable (systolic BP <90 mmHg). The criteria used for a positive TVU were presence of a cystic structure >4 cm, multitissue density structure, tubal dilation, uterine enlargement or mass, and extensive peritoneal fluid. In Switzerland, TVU is exclusively performed by gynaecologists. Therefore, all patients in need of a TVU have to be transferred to the gynaecology department or to a gynaecologist. Outside the hospital, patients and emergency medical services have to decide which department to approach since emergency physicians in Switzerland, Germany, and Austria do not see gynaecological and obstetric emergencies but have to consult a gynaecologist. This can be very time consuming and uncomfortable for the patient, who may have to be transferred between departments and even buildings as, in most major hospitals in Switzerland, departments of gynaecology and emergencies are located in different buildings. Due to this systemic limitation, most female patients whose RLQ abdominal pain is thought to be probably linked to a gynaecological problem end up being sent directly to the gynaecology department within the hospital, bypassing the ED. This may of course artificially reduce the number of patients with true gynaecological emergencies seen in our surgical ED, for whom TVU may have played an essential role in providing the correct diagnosis. This also means that some of those patients might have received a TVU and could have potentially met our inclusion criteria but were missed because they were sent to the gynaecology department.

## 3. Results

### 3.1. General Analysis

**Figure 1:**
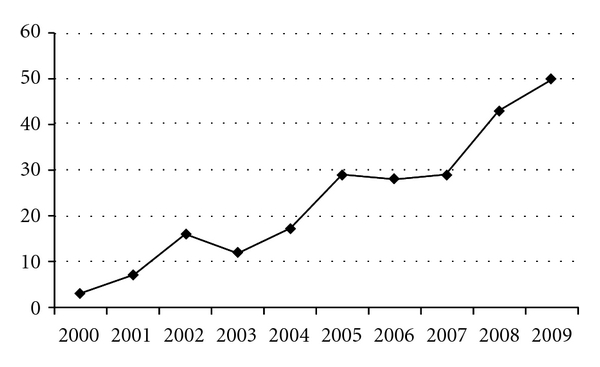
Number of patients undergoing a transvaginal ultrasound and meeting all study inclusion criteria, selected per year.

The mean age of the 224 patients included was 27.5 (range 16–53) years. While all patients suffered from RLQ abdominal pain (inclusion criteria), only 54.9% had rebound tenderness on physical examination and 32.5% Rovsing's sign—all details are summarized in Table 1. The number of patients undergoing TVU has increased in an almost linear fashion between 2000 and 2009 (Figure 1).

### 3.2. Patients with Negative TVU

**Figure 2:**
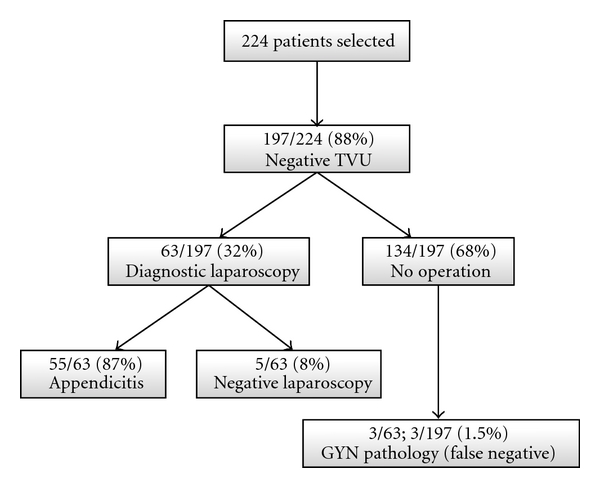
Patients with negative transvaginal ultrasound (TVU).

Of the 224 patients, 197 (87.9%) had a negative or an inconclusive TVU. 63 (32%) of these patients required diagnostic laparoscopy by the on-call general surgeon. Of these patients, 55 (87.3%) had appendicitis; three (5%) were found to have a gynecological pathology (one patient each with an uncomplicated ovarian cyst, a ruptured ovarian cyst, and an adnexitis), while five (8%) patients had no identified intra-abdominal pathology (Figure 2). The remaining 134 patients had a negative workup and were discharged within 12 hours of admission after spontaneous resolution of their symptoms. 

### 3.3. Patients with Positive TVU

**Figure 3:**
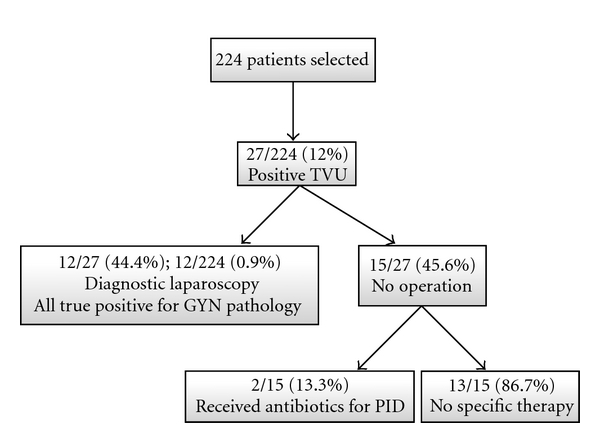
Patients with positive transvaginal ultrasound (TVU).

Only 27 (12.1%) patients were found to have a positive TVU. Of these, 12 underwent diagnostic laparoscopy, in which a gynaecological pathology was confirmed. The postoperative diagnoses of all patients needing a diagnostic laparoscopy (irrespective of the outcome of the emergency gynaecological examination) are summarized in Table 2. Only six patients had a true gynecological emergency (three ruptured ovarian cysts, one haemorrhagic necrosis of the right salpinx, one haemorrhagic corpus luteum, and one tuboovarian abscess). The remaining 15 patients with a positive TVU (functional cysts, uterine myomas, and nonspecific ovarian changes) did not require a surgical intervention and received no specific treatment, other than antibiotics for pelvic inflammatory disease in two patients (Figure 3).

## 4. Discussion

True gynaecological emergencies were very uncommon in our patient cohort. Only seven (3.1%) patients who presented to our ED with RLQ abdominal pain and in whom a TVU was carried out actually suffered from a gynaecological pathology necessitating immediate intervention. TVU was able to provide a correct diagnosis in all but one of these patients. This resulted in 100% specificity and 80% sensitivity with regard to detection of gynaecological pathologies, which compares well with the literature [5, 6]. Although classified as gynecologic emergencies, none of our patients had a life-threatening pathology.

The rate of TVU increased in a near linear fashion during the study period, with almost 44 TVU, performed in the first half of the study (2000–2004) and 180 performed in the second half (2005–2009). Although this increase could have affected the rate of negative laparoscopies performed by general surgeons, no statistical analysis was performed to study this effect as our study was purely descriptive. The linear increase in TVU rate seen in our data remains unexplained. We were unable to identify any factors or significant findings that could have affected the decision of the ED physician to request a TVU. There were no clinical rules or directives in effect in the department at the time to guide physicians in ordering a TVU. This increase could be explained by the escalation in medico-legal pressure or perhaps by a selection of more young and inexperienced ED physicians who ordered TVU as part of “defensive medicine” practice and asked for unnecessary tests and procedures as a protective measure. Furthermore, the majority of our patients (88%) had a negative TVU. These findings raise the question on the specific indications for emergency gynaecological consultations with TVU in NPF with RLQ abdominal pain. Our own experience shows that TVUs for patients admitted to our ED are usually requested without a specific algorithm and are more often requested as a means of excluding an underlying pathology, rather than of confirming a suspected gynaecological problem. A study by Gjelsteen et al. showed that TVU played a central role in the evaluation of nongravid patients with pelvic pain, as well as in the workup for ectopic pregnancies and adnexal masses [1]. The advantages of TVU also extend towards detection of abnormalities affecting the endometrium and myometrium, including foreign bodies, infection, fluid collections, hyperplasia, and neoplasia assessment [7]. Others have shown that TVU offered more accurate diagnostic information than conventional transabdominal ultrasound. Especially when used for evaluating pelvic masses or suspected ectopic gestations, TVU can be used as a diagnostic tool, complementary to the transabdominal technique [8–11]. The use of TVU has the advantage of increasing the diagnostic accuracy for nonemergency gynaecological pathologies such as symptomatic uterine myomas, complex ovarian cysts, and PID that could develop into life-threatening TOA or cause permanent damage to the tubes with infertility and which should prompt further outpatient workup and followup by a gynaecologist. In such cases, diagnostic TVU could possibly decrease the unnecessary use of CT scans and other costly and potentially harmful workup. The 12.1% rate of positive TVU workup prompts us to propose that, in addition to the pelvic exam that is indicated for any female with lower abdominal pain, a pelvic US and/or even a TVU should be considered in these patients as part of the basic workup.

As almost half the patients with a positive TVU had to undergo diagnostic laparoscopy, it may be that most women in enough pain to come to the ED for evaluation would be willing to undergo a pelvic exam and TVU if they knew that there was a chance of finding an explanation for their symptoms and of sparing them unnecessary surgery. While the advantages and benefits of TVU have been well documented, TVU remains a challenging and invasive procedure with its own limitations. Of all the types of diagnostic examinations performed, TVU remains one of the greatest challenges for radiology residents and faculty alike [12]. The spatial relationship between the transducer position and the image is still confusing for novices. Furthermore, TVU is an uncomfortable procedure for most NPF patients, with most preferring to be examined by a female sonographer and preferably a doctor, rather than a nurse or technician [4].

### 4.1. Limitations

Our findings are limited by the retrospective design of this study. There was no followup after discharge from the ED. The patients were discharged with instructions for elective followup in outpatient clinics or with their own family physicians. However, since we are contacted by the family physicians in case we have “missed” a diagnosis and since those patients who were discharged without a specific diagnosis from our ED are followed-up within 24–48 hours in our outpatient departments, we can presume that none of the patients discharged with a negative TVU was readmitted with a missed diagnosis.

 We limited our data analysis to NPF patients with RLQ admitted to our ED who needed a TVU. We were not able to evaluate data on patients who did not receive a TVU but who were found to have a gynaecological pathology, diagnosed either intra-operatively or postoperatively after admission to the general surgery department.

In addition, due to our inclusion criteria and the fact that we obtained our patients' database by query of our electronic medical record system, the filtered results only included patients with RLQ abdominal pain who underwent TVU. Thus, the overall percentage of female patients with RLQ pain or abdominal pain undergoing TVU is not reported, so any potential selection bias cannot be assessed. The fact that women can choose whether to go to the general ED or to be seen by the gynecological department for acute abdominal pain makes the 12.1% positive rate of TVU even more striking. It must be assumed that the rate of gynecological causes for RLQ pain would be even higher in a completely unselected patient group.

We also need to point that, unlike the US model, ED residency in Switzerland does not exist as a fully independent integrated residency. Residents spend 6–12 months in our department while training for a different specialty. Consultants are usually general surgeons or general physicians with a special interest in emergency medicine. These consultants work full time in the ED as is the case in the majority of countries where there is no board certification in emergency medicine. Thus, training in pelvic exams and TVU is not a normal practice for those physicians that work in our ED and is not an integrated part of the 6–12-month ED rotations so that TVU *can* only be performed by the gynaecologists.

## 5. Conclusion

Although TVU is a helpful tool for examining NPF patients with RLQ abdominal pain, improving early identification of gynaecological pathologies, and allowing for specific therapies, it could be perceived as an uncomfortable procedure if not performed by a physician who is familiar and well trained in its use. If a training program in pelvic examination and TVU could be introduced for the residents and rotating physicians in our ED, coupled to the provision of an ultrasound machine, this would help to increase the number of TVUs performed. In turn, this would enhance diagnosis by TVU and possibly spare the patients unnecessary surgery.

## Figures and Tables

**Table 1:** Study population clinical characteristics.

History and clinical examination	No. (%) of patients
*Previous history of*	
Sexually transmitted diseases	8 (3.6)
Abdominal surgery	10 (4.5)
Gynaecological surgery	32 (14.3)
Pregnancy (vaginal delivery)	54 (24.1)
Pregnancy (caesarean section)	22 (9.8)

*Clinical examination*	
RLQ abdominal tenderness	224 (100)
Rebound tenderness	123 (54.9)
Rovsing's sign	73 (32.6)
Psoas sign	53 (23.7)
Vaginal discharge	8 (3.6)
Cervical motion tenderness	21 (9.4)
Fundal tenderness	7 (3.1)
Cervical motion tenderness	21 (9.4)
Left adnexal tenderness—pelvic exam	17 (7.6)
Right adnexal tenderness—pelvic exam	29 (12.9)

**Table 2:** Postoperative diagnoses of patients requiring a diagnostic laparoscopy.

	Patients requiring a laparoscopy, *N* = 75 in Total	
	Positive transvaginal ultrasound (*N* = 12)	Negative transvaginal ultrasound^1^ (*N* = 63)
Gynaecological findings	Ruptured ovarian cyst* *N* = 3	Ruptured ovarian cyst (left side)* *N* = 1
	Nonruptured ovarian cyst *N* = 3	Nonruptured ovarian cyst *N* = 1
	Haemorrhagic necrosis, right salpinx* *N* = 1	Adnexitis *N* = 1
	Endometriosis *N* = 2	
	Right-sided tuboovarian abscess* *N* = 1	
	Uterine fibrosis^2^ *N* = 1	
	Haemorrhagic corpus luteum* *N* = 1	

Other findings		Appendicitis *N* = 55
		No abdominal pathology *N* = 5

^1^Indicates patients in whom diagnostic laparoscopy was performed by general surgeons following a negative transvaginal ultrasound.

^2^This patient underwent total laparoscopic hysterectomy without oophorectomy.

*Indicates a true gynecological emergency.
